# Neuroanatomical changes of ionotropic glutamatergic and GABAergic receptor densities in male mice modeling idiopathic and syndromic autism spectrum disorder

**DOI:** 10.3389/fpsyt.2023.1199097

**Published:** 2023-07-21

**Authors:** Leonardo Nardi, Stuti Chhabra, Petra Leukel, Dilja Krueger-Burg, Clemens J. Sommer, Michael J. Schmeisser

**Affiliations:** ^1^Institute of Anatomy, University Medical Center of the Johannes Gutenberg-University, Mainz, Germany; ^2^Focus Program Translational Neurosciences, University Medical Center of the Johannes Gutenberg-University Mainz, Mainz, Germany; ^3^Institute of Neuropathology, University Medical Center of the Johannes Gutenberg-University, Mainz, Germany

**Keywords:** ASD, autism, receptor autoradiography, ionotropic receptors, glutamate, GABA

## Abstract

Autism spectrum disorder (ASD) comprises a wide range of neurodevelopment conditions primarily characterized by impaired social interaction and repetitive behavior, accompanied by a variable degree of neuropsychiatric characteristics. Synaptic dysfunction is undertaken as one of the key underlying mechanisms in understanding the pathophysiology of ASD. The excitatory/inhibitory (E/I) hypothesis is one of the most widely held theories for its pathogenesis. Shifts in E/I balance have been proven in several ASD models. In this study, we investigated three mouse lines recapitulating both idiopathic (the BTBR strain) and genetic (*Fmr1* and *Shank3* mutants) forms of ASD at late infancy and early adulthood. Using receptor autoradiography for ionotropic excitatory (AMPA and NMDA) and inhibitory (GABA_A_) receptors, we mapped the receptor binding densities in brain regions known to be associated with ASD such as prefrontal cortex, dorsal and ventral striatum, dorsal hippocampus, and cerebellum. The individual mouse lines investigated show specific alterations in excitatory ionotropic receptor density, which might be accounted as specific hallmark of each individual line. Across all the models investigated, we found an increased binding density to GABA_A_ receptors at adulthood in the dorsal hippocampus. Interestingly, reduction in the GABA_A_ receptor binding density was observed in the cerebellum. Altogether, our findings suggest that E/I disbalance individually affects several brain regions in ASD mouse models and that alterations in GABAergic transmission might be accounted as unifying factor.

## Introduction

Autism Spectrum Disorder (ASD) is a complex neurodevelopmental condition involving altered social communication and presence of repetitive behaviour. Several other co-occurring conditions such as attention deficit hyperactivity disorder, depression, sleep disorder, epilepsy, anxiety, and intellectual disability are often associated with ASD (1). The prevalence of ASD has increased significantly in recent decades, with 1 in 100 children being affected across all socioeconomic, racial, and ethnic groups (2). Being a long-lasting condition, approximately 2% of the adult population is estimated to live with ASD (3). Although both genetic and environmental factors have been associated with ASD onset, its etiology still remains poorly understood. ASD is predominantly a heterogeneous disorder, majorly classified into syndromic and idiopathic forms. Syndromic cases are associated with clinically defined somatic and behavioral phenotypes. On the other hand, idiopathic forms have unknown etiology and account for the majority of ASD cases (4). However, synaptic dysfunctions remain a point of commonality among several disparate forms of autism (5).

Despite the neuroanatomical differences between humans and mice, some fundamental aspects of the neural mechanisms identified in animal models remain conserved across species and hence, translatable. Therefore, three well-renowned mouse strains characterized to study ASD features were chosen for this study. Black and tan brachyury (BTBR) mice are an inbred strain showing face validity for idiopathic ASD (6). Fragile X messenger ribonucleoprotein 1 (FMR1) is an mRNA binding protein mutated in fragile X syndrome, which is the most common cause of inherited intellectual disability and shares large degree of similarities in symptomatology with ASD. *Fmr1* knockout (KO) mice are the best characterized model to study fragile X syndrome. This model also shows promising behavioural and physiological features to be used as a validated model for ASD (7). SH3 and multiple ankyrin repeat domain 3 (SHANK3) is a key post-synaptic scaffolding protein, whose disruption is associated with the development of Phelan-McDermid Syndrome. *Shank3b* KO mice display key behavioral abnormalities associated with ASD (8).

Tight balance between excitatory and inhibitory synaptic transmission at neural circuits is crucial for normal brain development and function. Accordingly, shifts in the excitation/inhibition (E/I) balance have been implicated in the development and maintenance of ASD. In recent years, the theory of E/I imbalance in ASD has gained a lot of attention. It has been postulated that the autistic brain may be overactive because of a ‘signaling imbalance’ with too much excitatory signaling or too little inhibition at synaptic or circuit levels. This may also in part provide an explanation to the high propensity of people with ASD to develop seizures or epilepsy (9). Cumulative evidence now emerges to support the notion of E/I imbalances in various neurodevelopmental disorders including ASD in humans (10–15). Multiple factors such as synapse development, synaptic plasticity, intrinsic neuronal excitability, and intracellular signaling pathways play crucial roles in modulating E/I balance at cellular and circuit levels. However, it is important here to consider that the notion of “E/I balance” determining whether brain circuits are in homeostasis or not is vastly over simplified, since (A) microcircuits in different brain regions are not a unidirectional entity. They can be affected by different mixtures of excitation and inhibition inputs, (B) within a single microcircuit, different sources of excitation and inhibition affect different aspects of neuronal functions, and (C) brain compensatory response for the imbalance should be also considered. A multitude of factors has a critical role in differentially contributing to regulate individual synapses, thereby contributing to the E/I imbalance (16). Novel modulators directed at restoring the E/I balance by mostly targeting synaptic ionotropic excitatory and inhibitory receptors, are proving a valuable tool and paving way to clinical trials (17).

Ionotropic receptors are ligand-gated ion channels, made up of multiple subunits. GABA_A_ (γ-aminobutyric acid, type A) is one of the main inhibitory receptors at synapses. There are in total 19 known subunits, which differentially combine in heteropentamers. Different subunits and their combinations contribute to the regional and functional diversity of the receptor, being most commonly composed of two alpha, two beta‚ and one gamma subunits (18). NMDA and AMPA receptors belong to the ionotropic glutamate receptors family. Functionally active NMDA receptors are heterotetramers composed of two obligatory GluN1 subunits along with two GluN2 or GluN3 subunits. Four different GluN2 and two different GluN3 subunits exist, adding up to the complex regional and developmental composition of the receptor (19). AMPA receptors are heterotetrameric combinations of the subunits GluA1, GluA2, GluA3, and GluA4 and are expressed throughout the brain. Being a highly dynamic receptor, trafficking, insertion, and removal of the GluA subunits at the synaptic membrane thus play major role in determining the efficacy of synaptic transmission (20). In this study, we investigated the binding profile to ionotropic excitatory glutamatergic and inhibitory GABAergic receptors in an age and region dependent fashion in three mouse models of ASD. Taken together, we show a convergent increase in the GABA_A_ receptor binding density at adulthood in dorsal hippocampus (DH), whereas reduced GABA_A_ receptor binding density was observed in the cerebellum (Cer) concomitantly. Quantitative evaluation of GABA_A_, AMPA, and NMDA ionotropic receptor distribution will thus contribute to develop a better understanding towards underpinning the selective role of these receptors in alterations of E/I balance.

## Materials and methods

### Animals

BTBR (BTBR *T^+^ Itpr ^tf^*/J, stock #002282), C57BL6/J (stock #000664), *Fmr1* (B6.129P2-*Fmr1^tm1Cgr^*/J, stock #003025), and *Shank3b* (B6.129-*Shank3^tm2Gfng^*/J, stock #017688) mice were purchased from Jackson laboratories and housed in a pathogen-free facility with 12 h light/dark cycle, food, and water available *ad libitum. Fmr1^−/y^* (*Fmr1* KO) mice were generated by (21). *Shank3b* (B6.129-*Shank3^tm2Gfng^*/J) mice were generated by replacing exons 13–16 with a neomycin resistance cassette (8). BTBR, *Fmr1^−/y^* (*Fmr1* KO), and *Shank3b^−/−^* (*Shank3b* KO) were used as test animals. C57BL6/J mice were used as controls for BTBR mice, wildtype littermates for *Fmr1* KO and *Shank3b* KO. Breeding was approved by the local authorities. Since ASD shows a higher prevalence in male individuals, only male mice were used in the experiments (1). The number of animals tested in each experiment is reported in every figure legend and in the results section. All the experiments were performed according to guidelines of the central animal facility institution (TARC, Mainz University Medical Center) representing those of the German Animal Welfare Act and the European Directive 2010/63/EU for the protection of animals used for scientific purposes. Reporting was carried out according to the ARRIVE guidelines for reporting *in vivo* experiments.

### Tissue collection and processing

Mice were decapitated and brains were rapidly frozen in isopentane. They were further stored at −80°C until cutting. Brain slices were serially cut (20 μm thickness) in the coronal plane with a cryostat microtome (Leica, Germany). Slices containing Cer were cut similarly in the sagittal plane. The following bregma points were chosen for the analysis: 1.93 mm for the PFC, between 1.53 mm and 0.97 mm for DS and VS, −1.55 mm for the DH. Sagittal sections cut 0.72 mm lateral to the midline were considered for the Cer. For the location of the regions of interest, we referred to Paxinos and Franklin (22).

Totally, for each region of interest, 5 slices were collected and stored at −80°C until further histological and autoradiographic experiments. The first two slices were used for histological staining, the successive two were incubated with the [H^3^]-labeled ligands for AMPA, NMDA and GABA_A_ receptors. Slices containing Cer were incubated only with the [H^3^]-labeled ligands for GABA_A_ receptor. Indeed, as already shown, [^3^H]MK-801 yields no signal in the Cer at the concentrations used in this study (23).

### Histology

Hematoxylin–eosin staining was performed to help spatially localize the regions of interest on the autoradiograms. Briefly, the frozen brain slices were acclimatized at room temperature for 10 min. The slices were then incubated in acetone for 5 min, briefly air dried, and dipped in hematoxylin (Thermo Fisher) for 1 min. After washing in running water for 10 min, the slices were put for 10 s in Eosin Y (Thermo Fisher). Then, the slices were dehydrated in increasing ethanol concentrations (96 and 100%) each for 2 min. Finally, the slices were placed for 3 min in xylol, and cover slipped with Cytoseal XYL (Thermo Fisher). Pictures were scanned at 4× magnification with a Leica microscope (Leica, Germany), digitized and transferred to the MCID program.

### *In vitro* receptor autoradiography

The receptor binding density for AMPA, NMDA, and GABA_A_ receptors was adapted from the protocols described in (24). The tritiated ligands [^3^H]AMPA, [^3^H]MK-801, and [^3^H]Muscimol were purchased from PerkinElmer (Germany). AMPA is an agonist of the homonymous receptor, MK-801, also known as dizocilpine, is an uncompetitive antagonist of the NMDA receptor and Muscimol is an agonist of the GABA_A_ receptor. In the first step, the pre-incubation, endogenous ligands were washed off. In the following main incubation, the tritiated ligands were incubated both in the presence of a competitor, in order to determine the unspecific binding, and without it, in order to assess the total binding. Finally, the slices were rinsed. The slices incubated with [^3^H]AMPA were additionally dried with a warm air stream for 2 s and afterwards with a cold air stream. Slices incubated with [^3^H]MK-801 and [^3^H]Muscimol were dried with a cold air stream. A detailed description of the protocols used is reported in Table 1.

**Table 1:** Receptor binding protocols for the [^3^H] ligands with competitors (noted with *) and incubation conditions.

Receptor-[^3^H] ligand	Procedure	Incubation buffer	Time/Temperature
AMPA-[^3^H]AMPA	Pre-incubation	50 mM Trisacetat (pH 7.2)	3 × 10 min at 4°C
	Main incubation	50 mM Trisacetat (pH 7.2) + 100 mM KSCN + 10 nM [^3^H]AMPA + 10 M Quisquilate*	45 min at 4°C
	1st Rinsing	50 mM Trisacetat (pH 7.2)	3 × 4 s at 4°C
	2nd Rinsing	2.5% Glutaraldehyd in Acetone	2 × 2 s
NMDA-[^3^H]MK-801	Pre-incubation	50 mM Tris-HCl (pH 7.2) + 50 μM Glutamate	15 min at 4°C
	Main incubation	50 mM Tris-HCl (pH 7.2) + 50 μM Glutamate + 30 μM Glycin + 50 μM Spermidin + 5 nM [^3^H]MK-801 + 100 μM MK-801*	60 min at room temperature
	1st Rinsing	50 mM Tris-HCl (pH 7.2) + 50 μM Glutamate	2 × 5 min at 4°C
	2nd Rinsing	Distilled Water	2 × 5 min at 4°C
GABA_A_-[^3^H]Muscimol	Pre-incubation	50 mM Trisodium Citrate (pH 7.0)	3 × 5 min at 4°C
	Main incubation	50 mM Trisodium Citrate (pH 7.0) + 7.7 nM [^3^H]Muscimol + 10 mM GABA*	40 min at 4°C
	Rinsing	50 mM Trisodium Citrate (pH 7.0)	3 × 3 s at 4°C

### Image acquisition and analysis

**Figure 1:**
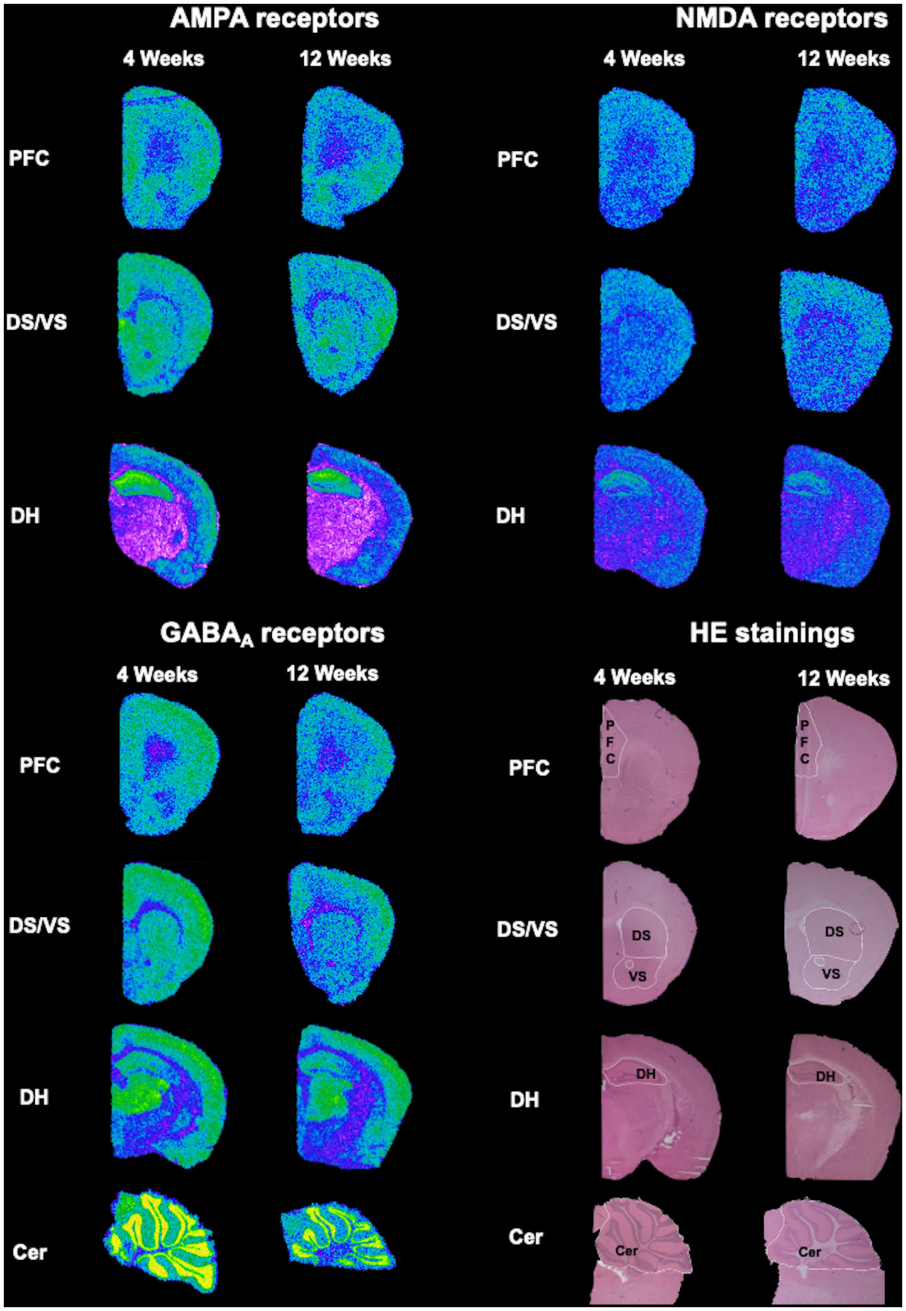
Exemplary overview of the autoradiograms and HE-stained sections. Slices stained with HE were used to trace the regions of interest (PFC, DS, VS, DH, and Cer), which were later overlayed on the respective autoradiograms for AMPA, NMDA and GABA_A_ receptors.

Image acquisition and analysis were performed as described in (25). [^3^H] plastic standards (Microscales^®^; Amersham, Freiburg, Germany) were exposed together with the tritium-labeled sections to a [^3^H]-sensitive film (Bio Max MR-1 Autoradiography Film, KODAKTM) for 12 ([^3^H]AMPA and [^3^H]Muscimol) and 15 ([^3^H]MK-801) weeks. The autoradiograms and the standards were scanned in equal lighting conditions with the digital CoolSNAP camera (Roper Scientific, Photometrics CoolSNAPTM cf., Ottobrunn/Munich Germany) and digitized with the MCID image analysis system (Imaging Research Inc., St. Catharines, Ontario, Canada). The standards were used to calculate the relationship between the gray values of the autoradiograms and the concentration of radioactivity. Total binding was calculated on the autoradiograms on both hemispheres in the regions of interest after tracing their boundary on the hematoxylin–eosin staining (Figure 1). The unspecific binding was consistently slightly above background signal or completely lacking. The value was then subtracted from the total binding. The binding values obtained from each ligand were used to calculate in the DH the E/I ratio as follows: (MK-801 + AMPA)/Muscimol (26).

### Statistical analysis

Statistical analysis was carried out with Prism (GraphPad, Version 9) and Microsoft Excel. Normal distribution of the data was assessed through the D’Agostino-Pearson test. Outliers were screened with the Rout test. Student’s multiple *t*-test was then performed. Raw *p*-values were then adjusted for multiple comparisons using the FDR correction method as described in (27). *p* < 0.05 was taken as threshold for statistical significance and results are shown as the mean ± SEM. The experiments were performed in a blinded manner and data are expressed as percentage of controls.

## Results

This dataset describes alterations in receptor binding densities to AMPA, NMDA, and GABA_A_ receptors in PFC, DH, DS, VS, and Cer. It was obtained by means of quantitative *in vitro* receptor autoradiography of 4- and 12 week-old BTBR, *Fmr1* KO and *Shank3b* KO mice. Representative HE-stained sections were used to trace the boundaries of the above-mentioned regions of interest, which were then overlapped on the autoradiograms for AMPA, NMDA, and GABA_A_ receptors prior to analysis (Figure 1).

**Figure 2:**
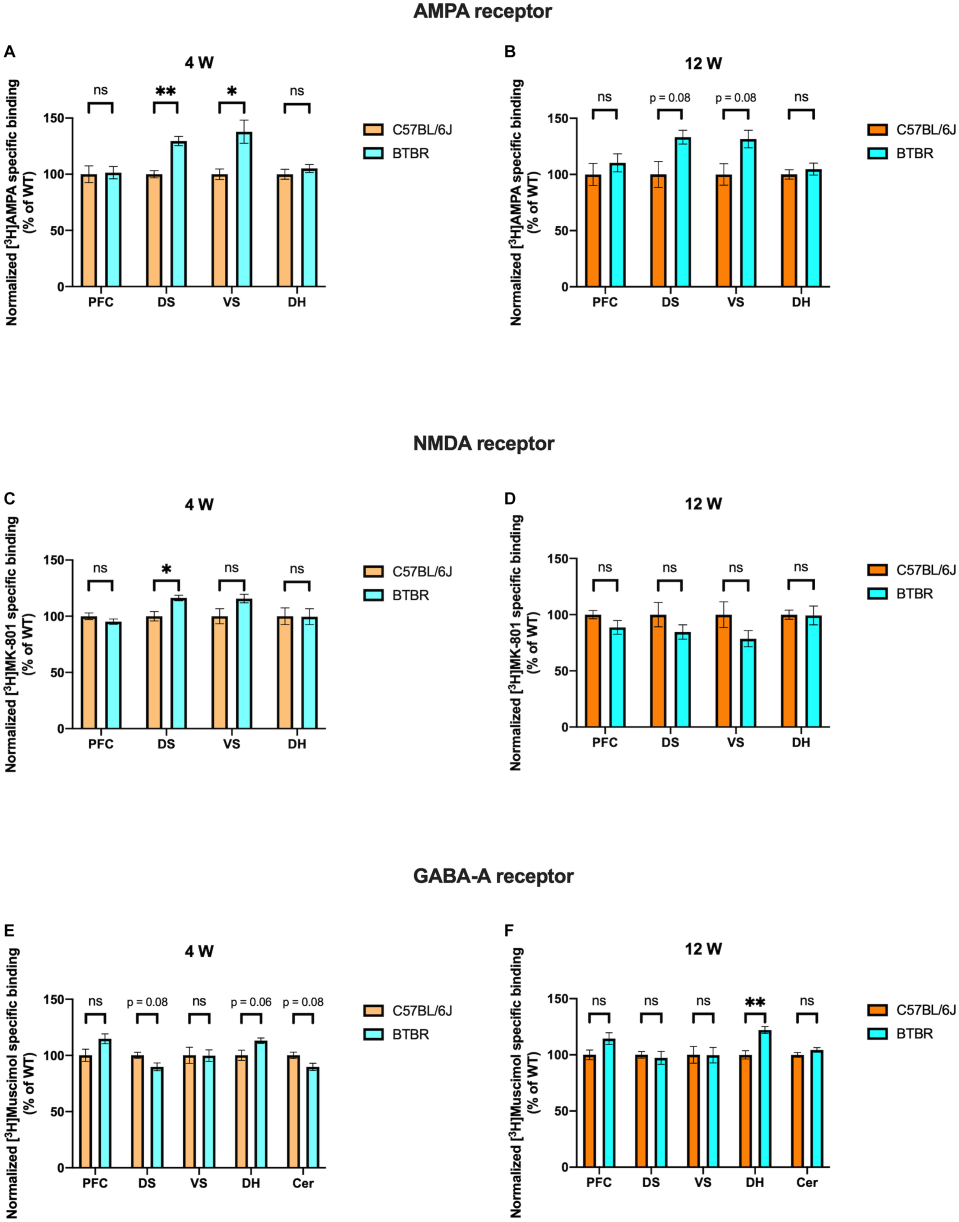
Bar charts representing mean and SEM of the receptor binding density in the BTBR line. Binding density to AMPA receptors at 4 weeks **(A)** PFC: C57BL6/J (*n* = 7), BTBR (*n* = 6), DS: C57BL6/J (*n* = 6), BTBR (*n* = 6), VS: C57BL6/J (*n* = 7), BTBR (*n* = 7), DH: C57BL6/J (*n* = 9), BTBR (*n* = 10). Binding density to AMPA receptors at 12 weeks **(B)** PFC: C57BL6/J (*n* = 9), BTBR (*n* = 9), DS: C57BL6/J (*n* = 10), BTBR (*n* = 10), VS: C57BL6/J (*n* = 10), BTBR (*n* = 10), DH: C57BL6/J (*n* = 9), BTBR (*n* = 10). Binding density to NMDA receptors at 4 weeks **(C)** PFC: C57BL6/J (*n* = 8), BTBR (*n* = 8), DS: C57BL6/J (*n* = 7), BTBR (*n* = 8), VS: C57BL6/J (*n* = 7), BTBR (*n* = 7), DH: C57BL6/J (*n* = 8), BTBR (*n* = 9). Binding density to NMDA receptors at 12 weeks **(D)** PFC: C57BL6/J (*n* = 8), BTBR (*n* = 10), DS: C57BL6/J (*n* = 10), BTBR (*n* = 10), VS: C57BL6/J (*n* = 10), BTBR (*n* = 10), DH: C57BL6/J (*n* = 10), BTBR (*n* = 10). Binding density to GABA_A_ receptors at 4 weeks **(E)** PFC: C57BL6/J (*n* = 8), BTBR (*n* = 9), DS: C57BL6/J (*n* = 8), BTBR (*n* = 9), VS: C57BL6/J (*n* = 7), BTBR (*n* = 8), DH: C57BL6/J (*n* = 9), BTBR (*n* = 9), Cer: C57BL6/J (*n* = 7), BTBR (*n* = 7). Binding density to GABA_A_ receptors at 12 weeks **(F)** PFC: C57BL6/J (*n* = 9), BTBR (*n* = 9), DS: C57BL6/J (*n* = 10), BTBR (*n* = 10), VS: C57BL6/J (*n* = 10), BTBR (*n* = 10), DH: C57BL6/J (*n* = 9), BTBR (*n* = 10), Cer: C57BL6/J (*n* = 9), BTBR (*n* = 9). Significant differences are indicated with asterisks (*p < 0.05 and **p < 0.01). Changes are represented as percentage of the mean of C57BL6/J mice.

In BTBR mice, receptor binding density to AMPA receptors was significantly increased at DS and VS at 4 weeks (DS, *p* = 0.003; VS, *p* = 0.025) and a similar tendency was observed at 12 weeks (DS, *p* = 0.087; VS, *p* = 0.087) of age. However, no significant alterations were observed in PFC (4 weeks, *p* = 0.980; 12 weeks, *p* = 0.612) and DH (4 weeks, *p* = 0.485; 12 weeks, *p* = 0.650) at both time points (Figures 2A,B). Interestingly, binding density to NMDA receptors also showed a significant increase in DS at 4 weeks (*p* = 0.025), but neither in VS (*p* = 0.102) nor in both striatal subregions at adulthood (DS, *p* = 0.383; VS, *p* = 0.299). There was no change in AMPA or NMDA receptor binding in PFC (4 weeks, *p* = 0.316; 12 weeks, *p* = 0.299) and DH (4 weeks, *p* = 0.980; 12 weeks, *p* = 0.979) at both time points (Figures 2C,D). Furthermore, receptor binding density to GABA_A_ receptors was not changed in PFC at both 4 weeks (*p* = 0.092) and 12 weeks (*p* = 0.158). In DS and Cer a tendency to reduced receptor binding density was found at 4 weeks (DS, *p* = 0.081; Cer, *p* = 0.081) but was not observed at adulthood (DS, *p* = 0.802; Cer, *p* = 0.299). GABA_A_ receptor binding density in VS showed no change at both time points (4 weeks, *p* = 0.980, 12 weeks, *p* = 0.979). Notably, a strong propensity to increased GABA_A_ receptor binding density was found at 4 weeks (*p* = 0.069), and it became significantly increased at 12 weeks (*p* = 0.003) of age in DH (Figures 2E,F). Taken together, both glutamatergic and GABAergic receptors demonstrate discrete changes in receptor binding densities. Still, we found the most prominent differences persisting during development in the binding to AMPA receptors in DS and VS (increased) and to GABA_A_ receptors in DH (increased).

**Figure 3:**
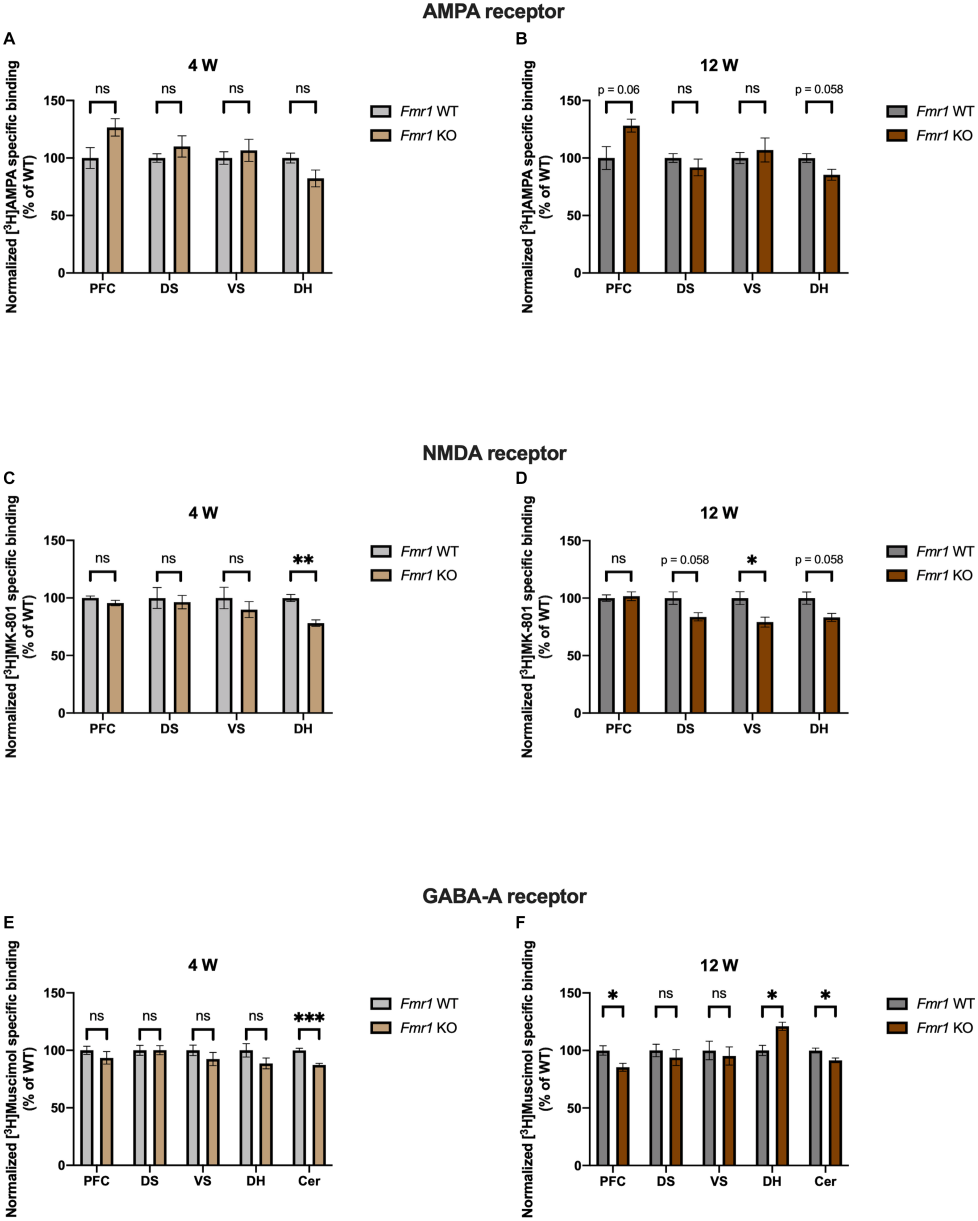
Bar charts representing mean and SEM of the receptor binding density in the *Fmr1* line. Binding density to AMPA receptors at 4  weeks **(A)** PFC: *Fmr1* WT (*n* = 8), *Fmr1* KO (*n* = 7), DS: *Fmr1* WT (*n* = 8), *Fmr1* KO (*n* = 8), VS: *Fmr1* WT (*n* = 9), *Fmr1* KO (*n* = 8), DH: *Fmr1* WT (*n* = 10), *Fmr1* KO (*n* = 9). Binding density to AMPA receptors at 12 weeks **(B)** PFC: *Fmr1* WT (*n* = 7), *Fmr1* KO (*n* = 6), DS: *Fmr1* WT (*n* = 7), *Fmr1* KO (*n* = 7), VS: *Fmr1* WT (*n* = 7), *Fmr1* KO (*n* = 6), DH: *Fmr1* WT (*n* = 10), *Fmr1* KO (*n* = 8). Binding density to NMDA receptors at 4 weeks **(C)** PFC: *Fmr1* WT (*n* = 7), *Fmr1* KO (*n* = 8), DS: *Fmr1* WT (*n* = 8), *Fmr1* KO (*n* = 8), VS: *Fmr1* WT (*n* = 8), *Fmr1* KO (*n* = 7), DH: *Fmr1* WT (*n* = 8), *Fmr1* KO (*n* = 6). Binding density to NMDA receptors at 12 weeks **(D)** PFC: *Fmr1* WT (*n* = 8), *Fmr1* KO (*n* = 8), DS: *Fmr1* WT (*n* = 6), *Fmr1* KO (*n* = 6), VS: *Fmr1* WT (*n* = 7), *Fmr1* KO (*n* = 6), DH: *Fmr1* WT (*n* = 10), *Fmr1* KO (*n* = 7). Binding density to GABA_A_ receptors at 4 weeks **(E)** PFC: *Fmr1* WT (*n* = 9), *Fmr1* KO (*n* = 8), DS: *Fmr1* WT (*n* = 9), *Fmr1* KO (*n* = 7), VS: *Fmr1* WT (*n* = 8), *Fmr1* KO (*n* = 6), DH: *Fmr1* WT (*n* = 10), *Fmr1* KO (*n* = 7), Cer: *Fmr1* WT (*n* = 9), *Fmr1* KO (*n* = 8). Binding density to GABA_A_ receptors at 12 weeks **(F)** PFC: *Fmr1* WT (*n* = 7), *Fmr1* KO (*n* = 8), DS: *Fmr1* WT (*n* = 6), *Fmr1* KO (*n* = 8), VS: *Fmr1* WT (*n* = 6), *Fmr1* KO (*n* = 8), DH: *Fmr1* WT (*n* = 10), *Fmr1* KO (*n* = 9), Cer: *Fmr1* WT (*n* = 6), *Fmr1* KO (*n* = 7). Significant differences are indicated with asterisks (*p < 0.05, **p < 0.01, and ***p < 0.001). Changes are represented as percentage of the mean of *Fmr1* WT mice.

*Fmr1* KO mice showed a propensity to increased binding density to AMPA receptors in PFC at 12 (*p* = 0.063) but not 4 weeks (*p* = 0.156). In DH we found the opposite, with a tendency to reduced receptor binding density at 12 (*p* = 0.059) but not at 4 weeks (*p* = 0.156). DS and VS showed no significant alterations at either time point (DS 4 weeks, *p* = 0.473; VS 4 weeks, *p* = 0.641; DS 12 weeks, *p* = 0.493; VS 12 weeks, *p* = 0.632) (Figures 3A,B). Binding density to NMDA receptors in PFC remained unchanged at both time points (4 weeks *p* = 0.373; 12 weeks, *p* = 0.729). In DS and VS, age dependent alterations are highlighted, with no change in receptor binding profile at 4 weeks (DS, *p* = 0.804; VS, *p* = 0.533) but reduced receptor binding densities at adulthood (DS, *p* = 0.058; VS, *p* = 0.049). DH showed reduced binding densities to NMDA receptors at both time points (4 weeks, *p* = 0.002; 12 weeks, *p* = 0.058) (Figures 3C,D). Regarding GABA_A_ receptors, binding density remained unaltered in PFC at 4 weeks (*p* = 0.473) but showed significant reduction at 12 weeks (*p* = 0.049). Similarly, no change was observed in DH at 4 weeks (*p* = 0.373), whereas it was strongly increased at adulthood (*p* = 0.025). Binding profile of DS and VS remained unchanged at both time points (DS 4 weeks, *p* = 0.984; VS 4 weeks, *p* = 0.473; DS 12 weeks, *p* = 0.632; VS 12 weeks, *p* = 0.729). Strikingly, binding density to GABA_A_ receptor was significantly reduced in Cer at both 4 (*p* = 0.0009) and 12 weeks (*p* = 0.049) (Figures 3E,F). Collectively, *Fmr1* KO mice showed several alterations, which remained consistent with development, such as NMDA binding in DH (decreased) and GABA_A_ binding in Cer (decreased).

Interesting similarities regarding GABA_A_ receptor binding density can be observed both in the BTBR and *Fmr1* KO mice. At 4 weeks, it was decreased in Cer (strong tendency in BTBR and significant change in *Fmr1* KO mice), whereas it was increased in DH at adulthood. This evidence highlights that the ionotropic neurotransmitter receptors analyzed might be discretely altered in different regions.

**Figure 4:**
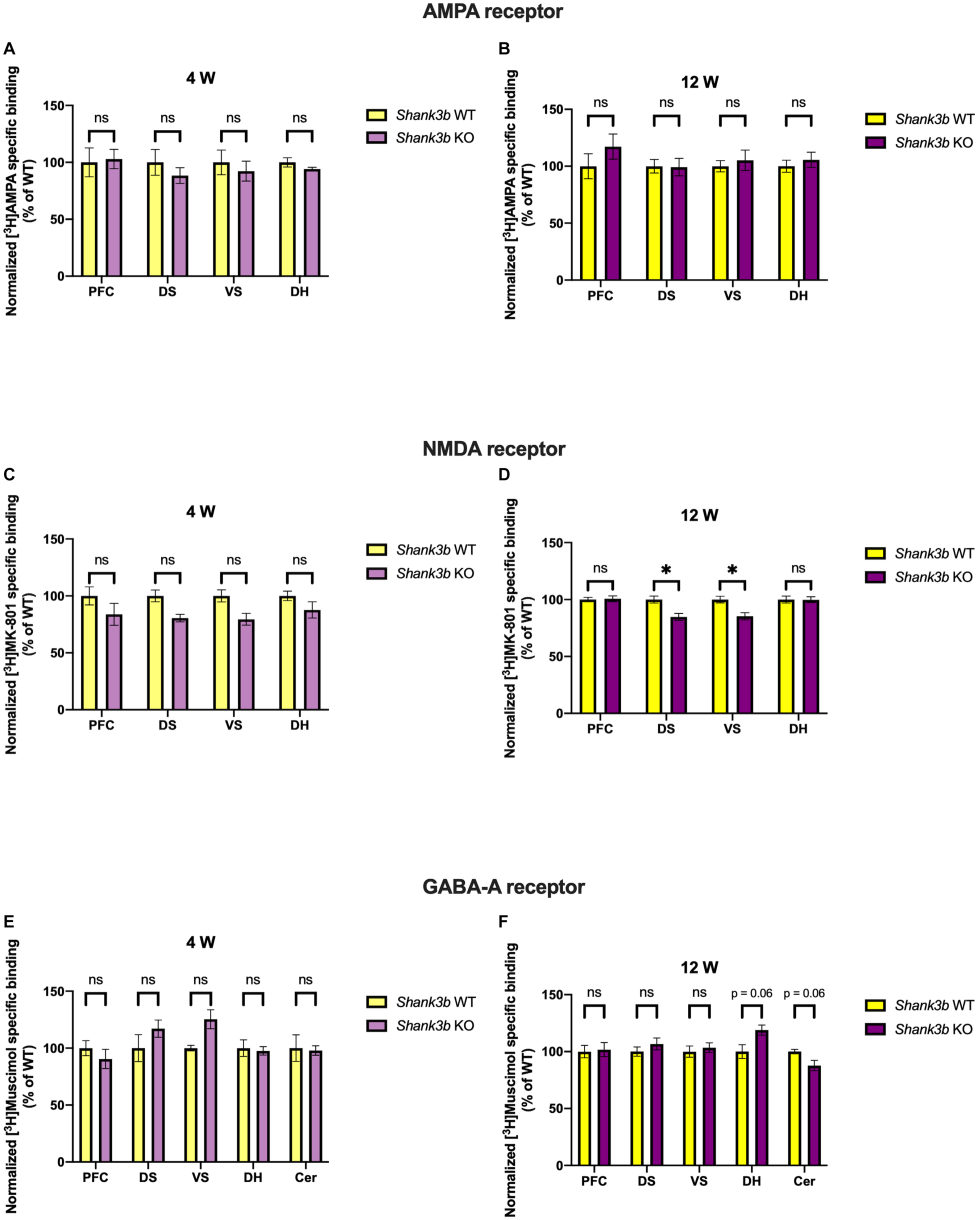
Bar charts representing mean and SEM of the receptor binding density in the *Shank3b* line. Binding density to AMPA receptors at 4 weeks **(A)** PFC: *Shank3b* WT (*n* = 6), *Shank3b* KO (*n* = 6), DS: *Shank3b* WT (*n* = 6), *Shank3b* KO (*n* = 6), VS: *Shank3b* WT (*n* = 6), *Shank3b* KO (*n* = 6), DH: *Shank3b* WT (*n* = 5), *Shank3b* KO (*n* = 5). Binding density to AMPA receptors at 12 weeks **(B)** PFC: *Shank3b* WT (*n* = 14), *Shank3b* KO (*n* = 11), DS: *Shank3b* WT (*n* = 15), *Shank3b* KO (*n* = 10), VS: *Shank3b* WT (*n* = 14), *Shank3b* KO (*n* = 11), DH: *Shank3b* WT (*n* = 14), *Shank3b* KO (*n* = 12). Binding density to NMDA receptors at 4 weeks **(C)** PFC: *Shank3b* WT (*n* = 5), *Shank3b* KO (*n* = 5), DS: *Shank3b* WT (*n* = 6), *Shank3b* KO (*n* = 5), VS: *Shank3b* WT (*n* = 6), *Shank3b* KO (*n* = 5), DH: *Shank3b* WT (*n* = 6), *Shank3b* KO (*n* = 6). Binding density to NMDA receptors at 12 weeks **(D)** PFC: *Shank3b* WT (*n* = 14), *Shank3b* KO (*n* = 12), DS: *Shank3b* WT (*n* = 14), *Shank3b* KO (*n* = 12), VS: *Shank3b* WT (*n* = 13), *Shank3b* KO (*n* = 12), DH: *Shank3b* WT (*n* = 14), *Shank3b* KO (*n* = 11). Binding density to GABA_A_ receptors at 4 weeks **(E)**; PFC: *Shank3b* WT (*n* = 6), *Shank3b* KO (*n* = 6), DS: *Shank3b* WT (*n* = 5), *Shank3b* KO (*n* = 6), VS: *Shank3b* WT (*n* = 4), *Shank3b* KO (*n* = 6), DH: *Shank3b* WT (*n* = 6), *Shank3b* KO (*n* = 6), Cer: *Shank3b* WT (*n* = 4), *Shank3b* KO (*n* = 6). Binding density to GABA_A_ receptors at 12 weeks **(F)** PFC: *Shank3b* WT (*n* = 15), *Shank3b* KO (*n* = 12), DS: *Shank3b* WT (*n* = 15), *Shank3b* KO (*n* = 12), VS: *Shank3b* WT (*n* = 15), *Shank3b* KO (*n* = 12), DH: *Shank3b* WT (*n* = 13), *Shank3b* KO (*n* = 12), Cer: *Shank3b* WT (*n* = 11), *Shank3b* KO (*n* = 11). Significant differences are indicated with asterisks (*p < 0.05). Changes are represented as percentage of the mean of *Shank3b* WT mice.

In *Shank3b* KO mice no change was found for AMPA receptor binding density for all regions analyzed, both at 4 (PFC, *p* = 0.852; DS, *p* = 0.583; VS, *p* = 0.772; DH, *p* = 0.442) and 12 weeks (PFC, *p* = 0.692; DS, *p* = 0.971; VS, *p* = 0.896; DH, *p* = 0.896), respectively (Figures 4A,B). Interestingly, binding density to NMDA receptors showed no change in DS and VS at 4 (DS, *p* = 0.146; VS, *p* = 0.146) but a significant reduction at 12 weeks (DS, *p* = 0.016; VS, *p* = 0.016). However, no significant alterations were observed at both time points in PFC (4 weeks, *p* = 0.442; 12 weeks, *p* = 0.971) and DH (4 weeks, *p* = 0.442; 12 weeks, *p* = 0.971) (Figures 4C,D). Concerning GABA_A_ receptors, at both time points considered, no change was noticed in PFC (4 weeks, *p* = 0.583; 12 weeks, *p* = 0.971), DS (4 weeks, *p* = 0.442; 12 weeks, *p* = 0.692), and VS (4 weeks, *p* = 0.188; 12 weeks, *p* = 0.896). Finally, no change was observed at 4 weeks in DH (*p* = 0.852) and Cer (*p* = 0.852), whereas at 12 weeks the receptor binding density showed strong tendencies to increase in DH (*p* = 0.068) and decrease in Cer (*p* = 0.068), respectively (Figures 4E,F). As already known from previous studies (28), knockdown of SHANK3 is associated to NMDA receptor reduction and hypofunction.

**Figure 5:**
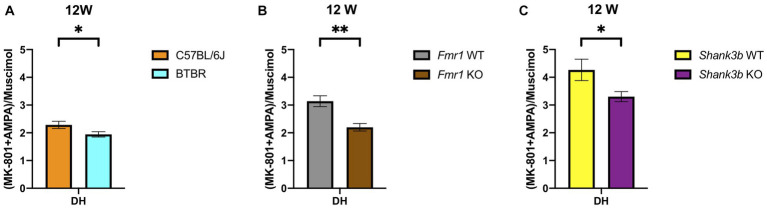
Bar charts representing mean and SEM of the receptor density-related E/I ratios from the DH of the three mouse lines analyzed in the study at 12 weeks of age. E/I ratio for at 12 weeks **(A)** DH: C57BL6/J (*n* = 8), BTBR (*n* = 10). E/I ratio for at 12 weeks **(B)** DH: *Fmr1* WT (*n* = 10), *Fmr1* KO (*n* = 6). E/I ratio for at 12 weeks **(C)** DH: *Shank3b* WT (*n* = 12), *Shank3b* KO (*n* = 11). Significant differences are indicated with asterisks (*p < 0.05, **p < 0.01).

Remarkably, increased GABA_A_ receptor binding density at adulthood in the DH was discovered as point of commonality among all the ASD mouse models analyzed in this study. This also leads to a disbalance in the binding to ionotropic excitatory and inhibitory receptors estimated through the calculation of the corresponding binding density-related E/I ratio (Figure 5). Moreover, in a region-specific fashion, GABA_A_ receptor binding density was reduced in Cer both in *Shank3b* (strong tendency) and *Fmr1* KO mice at adulthood.

## Discussion

In this study, we report for the first time the analysis of the binding density to the main ionotropic excitatory (AMPA and NMDA) and inhibitory (GABA_A_) receptors at the synapse by the means of receptor autoradiography in three different ASD mouse models. For the analysis, we chose two developmental time points corresponding to late infancy (4 weeks) and early adulthood (12 weeks). It has indeed been shown that synaptic proteins go through dynamical regulation during postnatal development (29). This consideration holds true also for AMPA (30), NMDA (19, 31, 32), and GABA_A_ (33–37) receptors. The study was limited to brain regions, in which the association to ASD is long known. Aware of the controversial definition of the murine PFC (38), we analyzed a region corresponding to the anterior cingulate, infralimbic and prelimbic cortex, as already previously shown (39). Frontal lobes in general, and PFC in particular, are known to play a role in complex social, cognitive, emotional and communicative skills (40). Str and NAcc are part of the basal ganglia and are functionally involved in the regulation of motor- and reward-motivated behavior (41). Although DH has been long associated to episodic memory and spatial orientations skills, recent acquisitions point at the involvement of this brain area in the development of the impaired social interaction phenotype typical of ASD (42). Finally, Cer is also embedded in the subcortical loops involved in the control of movement. Recent discoveries point out at the involvement of cerebellar projections to the emergence of social and cognitive impairment (43). Both macro- and microscopic alterations of the above-mentioned regions have been reported in ASD and have been hence selected for our screening (Figure 1) (44).

The E/I (excitation–inhibition) balance theory assumes that several psychiatric diseases, among which ASD, are due to a dysregulation of the excitatory and inhibitory factors existing at cellular, synaptic and circuit level, leading to a detrimental overall circuit activity (9, 16). Several factors contribute to the generation and maintenance of the E/I balance, such as glutamatergic and GABAergic ionotropic (AMPA, NMDA, GABA_A_) and metabotropic receptors, signaling pathways, intrinsic neuronal excitability, homeostatic synaptic plasticity, interneurons, and glial cells (45, 46). Notably, disrupting the E/I balance in mice has been associated to the onset of impairments in social interaction (47). Moreover, additional ASD mouse models not investigated in this study such as, for example, *Nf1* KO (48), *Cntnap4* KO (49), and *Tsc1* KO mice (50) showed altered E/I balance. In the present study, attention was restricted to a singular factor contributing to the E/I balance, namely the analysis of the ionotropic receptors. Alterations of the ionotropic glutamatergic and GABAergic receptors in ASD models and patients are in part already known and will be hence here discussed. We need to point out though, that there has been little use so far of receptor autoradiography to evaluate the brain receptor distributions in ASD models, making the comparison with other studies, using different methodological approaches, difficult. Having the BTBR strain a very strong face validity for ASD, a great number of drugs was tested in this line. Knowledge about the mechanisms leading to the typical phenotypes of this line is still largely limited. Impairments in glutamatergic neurotransmission were demonstrated in cortical synaptoneurosomes obtained from aged BTBR stimulated with potassium chloride (51). The use of AMPAKINE, positive modulators for AMPA receptors, in adult male and female BTBR mice led to improvement in social interaction and in learning and memory but not in the repetitive behavior (52). This is interesting to note, since we reported a significant increase of the AMPA binding density at 4 weeks and a strong tendency in the same direction at 12 weeks in the basal ganglia (Figures 2A,B), a region typically associated to the repetitive behavior phenotype, but not in the other regions considered. Increased D-aspartate, agonist of NMDA receptors, in PFC, hippocampus, and serum of BTBR, implicates alterations in the NMDA-related neurotransmission (53). Administration of D-cycloserine, a partial agonist of the NMDA receptor, led to an improvement of the impaired social interaction phenotype (54). Furthermore memantine, an NMDA receptor antagonist, could reduce the repetitive behavior phenotype (55). For the first time, we provided a detailed neuroanatomical mapping of the NMDA receptor binding density in the BTBR line, highlighting increased binding at 4 weeks in the DS (Figure 2C). Moreover, increased levels of glutamate, glutamine and GABA_A_ were found in the Str of BTBR mice by the means of proton magnetic resonance spectroscopy (12). Reduced GABA_A_ mediated inhibitory transmission in the BTBR hippocampus at 3 weeks has been reported. Administration of L-838,417, a partial agonist specific for the GABA_A_ receptor subunits α2 and α3, proved efficacious in reducing the social impairment, whereas zolpidem, an α1 selective positive allosteric modulator, aggravated it (56). We showed at 12 weeks increased GABA_A_ receptor binding density in the DH (Figure 2F). A similar shift was observed also at 4 weeks (Figure 2E). These two observations should not be seen in contrast to each other since mutations in the scaffold protein gephyrin can lead to reduced GABAergic transmission in the presence of unchanged overall membrane expression (57, 58). Reduction of GABAergic transmission was moreover reported in the insular cortex of BTBR mice, resulting in defective multisensory integration. The deficit could be rescued with the application of diazepam, agonist at the benzodiazepine binding site of the GABA_A_ receptor (59). Successful application of diazepine in ameliorating the BTBR phenotype had already been reported (60). Other studies also showed the effects of drugs acting on the GABA_A_-related system in male and female BTBR mice, such as gaboxadol, a potent GABA_A_ agonist, (61) and ganaxolone, a positive GABA_A_ allosteric modulator (62). In another study, a selective positive allosteric modulator of GABA_A_ receptor proved effective on adult male BTBR mice (63). Fragile X syndrome is the most common form of genetic intellectual disability and autism (64). The huge amount of evidence pertaining the synaptic function in the *Fmr1* KO model is therefore not surprising. In one report, reduced AMPA receptor subunit GluA1 was found in the cortex but not in the hippocampus and in the Cer of *Fmr1* KO mice, whereas no changes in NMDA receptor subunits were detected (65). Further evidence showed reduced levels of GluA1 phosphorylated at the serine 831 in the hippocampal dentate gyrus and cornu ammonis (66, 67). The phosphorylation of this amino acid is crucial for displaying normal long-term potentiation and long-term depression (68). In our hands, we found brain region-specific modifications, i.e., AMPA receptor binding density tended to increase in the PFC and to decrease in DH at 12 weeks in the *Fmr1* KO mice (Figure 3B). In another report, reduced GluN1, GluN2a, and GluN2b were detected in the PFC of *Fmr1* KO mice (69). In the dentate gyrus, impaired neurotransmission mediated from NMDA receptor was registered in multiple studies (66, 70–72). In one of them, moreover, the NMDA receptor subunits GluN1, GluN2a, and GluN2b were also found reduced in the dentate gyrus (66). We also found a consistent decrease of NMDA binding density at 4 weeks and a similar pattern at 12 weeks in the DH of *Fmr1* KO mice. Moreover, at 12 weeks significant reduction was also registered in DS and VS, whereas in the PFC no change was revealed. The differences found may be due to the dissimilarities among the experimental procedures performed in this and other studies (Figures 3C,D). Evidence about alterations of the GABAergic metabolism and neurotransmission is abundant (for a complete overview please refer to (73, 74)). mRNA levels of several GABA_A_ receptor subunits were found reduced in the cortex, but not in the hippocampus of *Fmr1* KO mice at 8–12 weeks (75). At 10 weeks of age, mRNA coding for several subunits of the GABA_A_ receptor were found reduced both in cortex and Cer (76). Analysis of full brain homogenates from *Fmr1* KO mice revealed, moreover, a tight temporal regulation, i.e., multiple GABA_A_ receptor subunits dysregulated at postnatal days 5 and 12 but not at early adulthood (77). In a previous report, the β subunit of the GABA_A_ receptor was found reduced in cortex, hippocampus, brainstem, and diencephalon but not in Cer of *Fmr1* KO mice at 8 weeks (78). Both α2 and β1 GABA_A_ subunits were found reduced at mRNA and protein level in the hippocampus of animals at postnatal day 22 (79). The striking convergence among the different studies, is the reduction of the δ subunit of the GABA_A_ receptor (76, 77, 79, 80). GABA_A_ receptors, which contain the δ subunit, are only 5% of the total, are located peri- or extrasynaptically and mediate tonic inhibition (18, 81). In this study, the ligand [^3^H]Muscimol was used. It binds to the GABA binding site of the GABA_A_ receptor, which is to be found between the alpha and beta subunits. Hence, in the present study nothing can be inferred about the δ subunit. Moreover, at 4 weeks only GABA_A_ binding density in the Cer was strongly reduced (confirming the trend observed in the literature) (Figure 3E), whereas at 12 weeks GABA_A_ binding density showed region specific changes, being reduced in PFC and Cer and increased in DH (Figure 3F). Subsequently, drugs targeting GABA_A_ receptors such as benzodiazepines, ganaxolone and gaboxadol have been employed successfully in mice models of fragile X syndrome (82, 83).

Mutations in *SHANK3* account for up to 0.7% of cases of ASD and a multitude of mouse models have been generated so far (84, 85). Hence, evidence available in the literature does not always derive from the same mouse model we used. By the means of cell surface biotinylation assay, reduced AMPA and NMDA receptor subunits were detected in the *Shank3αβ* KO model at 3–6 months in thalamus, hippocampus and striatum (86). In male and female *Shank3^e4–9^* KO mice, reduced GluA1 and GluN2a levels were registered (87). GluA2, GluN2a, and GluN2b were also found reduced in fractions obtained from the postsynaptic density of *Shank3b* KO mice (8). Interestingly, the use of an AMPAKINE and of D-cycloserine in *Shank3b* KO mice proved of limited efficacy (61). Although we found no changes regarding AMPA receptor binding density at both time points (Figures 4A,B), receptor binding to the NMDA receptor was reduced in DS and VS at adulthood (Figure 4D). Alterations of GABAergic markers were identified in pups and adult *Shank3b* KO mice (88). A recent study showed no significant changes regarding the binding availability to the benzodiazepine binding site of the GABA_A_ receptor both *in-vivo* on ASD patients (via PET scan) and *in-vitro* on ASD mouse models, among them *Shank3b* KO mice (via receptor autoradiography) (89). Of relevance, regarding binding density to the GABA_A_ receptor, we found a tendency to increase in the DH and decrease in the Cer at adulthood (Figure 4F). Discrepancies from the studies reported above might depend upon the different ligands utilized. Finally, we intend to highlight the convergent increase at adulthood in DH of the GABA_A_ receptor binding densities in all the lines investigated (Figures 2F, 3F, 4F) and reduction in Cer in *Fmr1* KO and *Shank3b* KO lines (Figures 3F, 4F). The increased binding density to GABA_A_ also affects the receptor density-related balance between excitation and inhibition in the DH at adulthood (Figure 5).

Several lines of evidence deriving from human research point at a decisive involvement of the GABAergic system in ASD. Molecular studies revealed a downregulation of GABA_A_ receptor subunits in parts of the PFC and Cer (90–92). We also showed reduced binding density to the GABA_A_ receptor in the Cer (Figures 2E, 3E, 3F, 4F). Receptor autoradiography in parts of the PFC from ASD individuals, revealed reduced binding density to the GABA and benzodiazepine binding sites (93, 94), mirroring in part our results (Figure 3F). In the hippocampus of ASD patients, binding density to the benzodiazepine binding site was reduced (95). In another study, [^3^HMuscimol] binding was reduced in the pyramidal layer of CA1 but not changed in the remaining ones (96). Even if in this study the same ligand was used, values were measured on the whole DH as already performed by (97) and not on the individual layers. *In-vivo* PET studies also revealed reduced binding to the benzodiazepine binding site of the GABA_A_ receptor in patients affected by fragile X syndrome in one portion of the PFC (98), exactly as observed in the *Fmr1* KO mice at 12 weeks (Figure 3F). The generalized binding throughout the brain to receptors α1 and α5 of the GABA_A_ receptor was found reduced in ASD patients (99). Finally, a SPECT study in individuals with ASD showed a reduced accumulation of a radioactive ligand binding to the benzodiazepine binding site of the GABA_A_ receptor in the superior and medial frontal cortex (100). The abundant evidence available about disorders of the E/I balance in ASD is at the root of the numerous pharmacological attempts directed at its modulation (17, 101).

A limitation of the technical approach used in this study consists in the lack of cell specificity. Moreover, the results reported indicate the percentual change of receptors available, but nothing can be inferred about the functional state. In recent years, a growing body of evidence has put in relation altered inhibitory neurotransmission in ASD with parvalbumin (PV)-positive interneurons. Briefly, they are a class of GABAergic cortical and hippocampal interneurons expressing the calcium-binding protein parvalbumin and fine-tuning the E/I balance in the brain (102). Reports from human brains highlight region-specific changes in the number or density of PV-positive interneurons, being increased in the DH (103) and decreased in the PFC (104). Mice devoid of PV recapitulate all the typical hallmarks of ASD (105). Interestingly, all the ASD models investigated in this study show region-specific alterations of PV-positive interneurons (106–108).

Future studies should be directed at investigating the role of the E/I imbalance (109) or PV-positive cells (110). Taken together, our study highlights developmental and region-specific alterations of the ionotropic receptors landscape in ASD mouse models. We believe that *in-vitro* approaches such as patient-derived induced pluripotent stem cells, organoids and assembloids can further pave the way in both modelling neuropsychiatric conditions and testing potential drugs acting on these membrane receptors.

## Data availability statement

The raw data supporting the conclusions of this article will be made available by the authors, without undue reservation.

## Ethics statement

Ethical review and approval was not required for the animal study because organ removal from mice for scientific purpose (the brain in this study) does not require approval by an ethics committee in Germany.

## Author contributions

SC, LN, PL, CJS, and MJS planned the autoradiographic experiments. SC, LN, and PL conducted the autoradiographic experiments and analyzed the data. SC and LN drafted the manuscript. PL, DK-B, CJS, and MJS critically revised and edited the manuscript. All authors contributed to the article and approved the submitted version.

## Funding

LN was supported by an internal grant of the University Medical Center, Mainz (Stufe I). MJS was supported by the German Research Foundation (DFG, Collaborative Research Center 1080, Project B10) and the Werner Reichenberger Foundation. DK-B was supported by the Heisenberg program of the DFG (grant KR 5329/1-1).

## Conflict of interest

The authors declare that the research was conducted in the absence of any commercial or financial relationships that could be construed as a potential conflict of interest.

## Publisher’s note

All claims expressed in this article are solely those of the authors and do not necessarily represent those of their affiliated organizations, or those of the publisher, the editors and the reviewers. Any product that may be evaluated in this article, or claim that may be made by its manufacturer, is not guaranteed or endorsed by the publisher.

## Figures and Tables

**Table:** 

[^3^H]	tritium
AMPA	α-amino-3-hydroxy-5-methyl-4-isoxazolepropionic acid
ASD	autism spectrum disorders
Cer	cerebellum
BTBR	black and tan brachyury
DH	dorsal hippocampus
DS	dorsal striatum
E/I	excitation–inhibition
FMR1	fragile X messenger ribonucleoprotein 1
GABA	γ-Aminobutyric acid
KO	knockout
NMDA	N-methyl-D-aspartate
PV	parvalbumin
PFC	prefrontal cortex
SEM	standard error of the mean
SHANK	SH3 and multiple ankyrin repeat domains protein 3
VS	ventral striatum
VTA	ventral tegmental area
WT	wildtype.

## References

[1] Lord C Brugha TS Charman T Cusack J Dumas G Frazier T. Autism spectrum disorder. Nat Rev Dis Primers. (2020) 6:5. doi: 10.1038/s41572-019-0138-4, PMID: 31949163 PMC8900942

[2] Zeidan J Fombonne E Scorah J Ibrahim A Durkin MS Saxena S. Global prevalence of autism: a systematic review update. Autism Res. (2022) 15:778–90. doi: 10.1002/aur.2696, PMID: 35238171 PMC9310578

[3] Dietz PM Rose CE McArthur D Maenner M. National and State estimates of adults with autism Spectrum disorder. J Autism Dev Disord. (2020) 50:4258–66. doi: 10.1007/s10803-020-04494-4, PMID: 32390121 PMC9128411

[4] Casanova MF Casanova EL Frye RE Baeza-Velasco C LaSalle JM Hagerman RJ. Editorial: secondary vs. idiopathic autism. Front Psych. (2020) 11:297. doi: 10.3389/fpsyt.2020.00297, PMID: 32346372 PMC7171716

[5] Carroll L Braeutigam S Dawes JM Krsnik Z Kostovic I Coutinho E. Autism spectrum disorders: multiple routes to, and multiple consequences of, abnormal synaptic function and connectivity. Neuroscientist. (2021) 27:10–29. doi: 10.1177/1073858420921378, PMID: 32441222 PMC7804368

[6] Meyza KZ Blanchard DC. The BTBR mouse model of idiopathic autism - current view on mechanisms. Neurosci Biobehav Rev. (2017) 76:99–110. doi: 10.1016/j.neubiorev.2016.12.037, PMID: 28167097 PMC5403558

[7] Bernardet M Crusio WE. Fmr1 KO mice as a possible model of autistic features. ScientificWorldJournal. (2006) 6:1164–76. doi: 10.1100/tsw.2006.220, PMID: 16998604 PMC5917219

[8] Peca J Feliciano C Ting JT Wang W Wells MF Venkatraman TN. Shank3 mutant mice display autistic-like behaviours and striatal dysfunction. Nature. (2011) 472:437–42. doi: 10.1038/nature09965, PMID: 21423165 PMC3090611

[9] Rubenstein JL Merzenich MM. Model of autism: increased ratio of excitation/inhibition in key neural systems. Genes Brain Behav. (2003) 2:255–67. doi: 10.1034/j.1601-183x.2003.00037.x, PMID: 14606691 PMC6748642

[10] Chattopadhyaya B Cristo GD. GABAergic circuit dysfunctions in neurodevelopmental disorders. Front Psych. (2012) 3:51. doi: 10.3389/fpsyt.2012.00051, PMID: 22666213 PMC3364508

[11] Eltokhi A Santuy A Merchan-Perez A Sprengel R. Glutamatergic dysfunction and synaptic ultrastructural alterations in schizophrenia and autism Spectrum disorder: evidence from human and rodent studies. Int J Mol Sci. (2020) 22:59. doi: 10.3390/ijms22010059, PMID: 33374598 PMC7793137

[12] Horder J Petrinovic MM Mendez MA Bruns A Takumi T Spooren W. Glutamate and GABA in autism spectrum disorder-a translational magnetic resonance spectroscopy study in man and rodent models. Transl Psychiatry. (2018b) 8:106. doi: 10.1038/s41398-018-0155-1, PMID: 29802263 PMC5970172

[13] Manyukhina VO Prokofyev AO Galuta IA Goiaeva DE Obukhova TS Schneiderman JF. Globally elevated excitation–inhibition ratio in children with autism spectrum disorder and below-average intelligence. Mol Autism. (2022) 13:20. doi: 10.1186/s13229-022-00498-2, PMID: 35550191 PMC9102291

[14] Port RG Oberman LM Roberts TP. Revisiting the excitation/inhibition imbalance hypothesis of ASD through a clinical lens. Br J Radiol. (2019) 92:20180944. doi: 10.1259/bjr.20180944, PMID: 31124710 PMC6732925

[15] Zhao H Mao X Zhu C Zou X Peng F Yang W. GABAergic system dysfunction in autism spectrum disorders. Front Cell Dev Biol. (2021) 9:781327. doi: 10.3389/fcell.2021.781327, PMID: 35198562 PMC8858939

[16] Sohal VS Rubenstein JLR. Excitation–inhibition balance as a framework for investigating mechanisms in neuropsychiatric disorders. Mol Psychiatry. (2019) 24:1248–57. doi: 10.1038/s41380-019-0426-0, PMID: 31089192 PMC6742424

[17] Canitano R Palumbi R. Excitation/inhibition modulators in autism Spectrum disorder: current clinical research. Front Neurosci. (2021) 15:753274. doi: 10.3389/fnins.2021.753274, PMID: 34916897 PMC8669810

[18] Rudolph U Knoflach F. Beyond classical benzodiazepines: novel therapeutic potential of GABAA receptor subtypes. Nat Rev Drug Discov. (2011) 10:685–97. doi: 10.1038/nrd3502, PMID: 21799515 PMC3375401

[19] Paoletti P Bellone C Zhou Q. NMDA receptor subunit diversity: impact on receptor properties, synaptic plasticity and disease. Nat Rev Neurosci. (2013) 14:383–400. doi: 10.1038/nrn3504, PMID: 23686171

[20] Henley JM Wilkinson KA. Synaptic AMPA receptor composition in development, plasticity, and disease. Nat Rev Neurosci. (2016) 17:337–50. doi: 10.1038/nrn.2016.37, PMID: 27080385

[21] Bakker CE Verheij C Willemsen R Vanderhelm R Oerlemans F Vermey M. Fmr1 knockout mice - a model to study fragile-X mental-retardation. Cells. (1994) 78:23–33. doi: 10.1016/0092-8674(94)90569-X 8033209

[22] Paxinos G Franklin KBJ. Paxinos and Franklin’s the mouse brain in stereotaxic coordinates. 5th Edn, San Diego: Elsevier Academic Press (2019).

[23] Sakurai SY Cha JH Penney JB Young AB. Regional distribution and properties of [^3^H]MK-801 binding sites determined by quantitative autoradiography in rat brain. Neuroscience. (1991) 40:533–43. doi: 10.1016/0306-4522(91)90139-f, PMID: 2027471

[24] Frauenknecht K Plaschke K Sommer C. Transient oligemia is associated with long-term changes in binding densities of cortical inhibitory GABAA receptors in the rat brain. Brain Res. (2009) 1271:95–102. doi: 10.1016/j.brainres.2009.03.028, PMID: 19328192

[25] Mammele S Frauenknecht K Sevimli S Diederich K Bauer H Grimm C. Prevention of an increase in cortical ligand binding to AMPA receptors may represent a novel mechanism of endogenous brain protection by G-CSF after ischemic stroke. Restor Neurol Neurosci. (2016) 34:665–75. doi: 10.3233/RNN-150543, PMID: 26410211

[26] Sommer C Fahrner A Kiessling M. [^3^H]muscimol binding to gamma-aminobutyric acid(a) receptors is upregulated in CA1 neurons of the gerbil hippocampus in the ischemia-tolerant state. Stroke. (2002) 33:1698–705. doi: 10.1161/01.str.0000016404.14407.77, PMID: 12053014

[27] Rosner B. Fundamentals of biostatistics. Cengage Learning, Inc (2011).

[28] Duffney LJ Wei J Cheng J Liu W Smith KR Kittler JT. Shank3 deficiency induces NMDA receptor hypofunction via an actin-dependent mechanism. J Neurosci. (2013) 33:15767–78. doi: 10.1523/JNEUROSCI.1175-13.2013, PMID: 24089484 PMC3787498

[29] Gonzalez-Lozano MA Klemmer P Gebuis T Hassan C van Nierop P van Kesteren RE. Dynamics of the mouse brain cortical synaptic proteome during postnatal brain development. Sci Rep. (2016) 6:35456. doi: 10.1038/srep35456, PMID: 27748445 PMC5066275

[30] Blair MG Nguyen NN Albani SH L’Etoile MM Andrawis MM Owen LM. Developmental changes in structural and functional properties of hippocampal AMPARs parallels the emergence of deliberative spatial navigation in juvenile rats. J Neurosci. (2013) 33:12218–28. doi: 10.1523/JNEUROSCI.4827-12.2013, PMID: 23884930 PMC4471169

[31] Monyer H Burnashev N Laurie DJ Sakmann B Seeburg PH. Developmental and regional expression in the rat brain and functional properties of four NMDA receptors. Neuron. (1994) 12:529–40. doi: 10.1016/0896-6273(94)90210-0, PMID: 7512349

[32] Watanabe M Inoue Y Sakimura K Mishina M. Developmental changes in distribution of NMDA receptor channel subunit mRNAs. Neuroreport. (1992) 3:1138–40. doi: 10.1097/00001756-199212000-00027, PMID: 1493227

[33] Fritschy JM Paysan J Enna A Mohler H. Switch in the expression of rat GABAA-receptor subtypes during postnatal development: an immunohistochemical study. J Neurosci. (1994) 14:5302–24. doi: 10.1523/JNEUROSCI.14-09-05302.1994, PMID: 8083738 PMC6577100

[34] Huntsman MM Munoz A Jones EG. Temporal modulation of GABA(a) receptor subunit gene expression in developing monkey cerebral cortex. Neuroscience. (1999) 91:1223–45. doi: 10.1016/s0306-4522(98)00713-1, PMID: 10391431

[35] Laurie DJ Seeburg PH Wisden W. The distribution of 13 GABAA receptor subunit mRNAs in the rat brain. II. Olfactory bulb and cerebellum. J Neurosci. (1992a) 12:1063–76. doi: 10.1523/JNEUROSCI.12-03-01063.1992, PMID: 1312132 PMC6576040

[36] Laurie DJ Wisden W Seeburg PH. The distribution of thirteen GABAA receptor subunit mRNAs in the rat brain. III. Embryonic and postnatal development. J Neurosci. (1992b) 12:4151–72. doi: 10.1523/JNEUROSCI.12-11-04151.1992, PMID: 1331359 PMC6576006

[37] Wisden W Laurie DJ Monyer H Seeburg PH. The distribution of 13 GABAA receptor subunit mRNAs in the rat brain. I. Telencephalon, diencephalon, mesencephalon. J Neurosci. (1992) 12:1040–62. doi: 10.1523/JNEUROSCI.12-03-01040.1992, PMID: 1312131 PMC6576059

[38] Laubach M Amarante LM Swanson K White SR. What, if anything, is rodent prefrontal cortex? eNeuro. (2018) 5:ENEURO.0315–18.2018. doi: 10.1523/ENEURO.0315-18.2018, PMID: 30406193 PMC6220587

[39] Luo Y Xiao Q Wang J Jiang L Hu M Jiang Y. Running exercise protects oligodendrocytes in the medial prefrontal cortex in chronic unpredictable stress rat model. Transl Psychiatry. (2019) 9:322. doi: 10.1038/s41398-019-0662-8, PMID: 31780641 PMC6882819

[40] Courchesne E Pierce K. Why the frontal cortex in autism might be talking only to itself: local over-connectivity but long-distance disconnection. Curr Opin Neurobiol. (2005) 15:225–30. doi: 10.1016/j.conb.2005.03.001, PMID: 15831407

[41] Fuccillo MV. Striatal circuits as a common node for autism pathophysiology. Front Neurosci. (2016) 10:27. doi: 10.3389/fnins.2016.00027, PMID: 26903795 PMC4746330

[42] Banker SM Gu X Schiller D Foss-Feig JH. Hippocampal contributions to social and cognitive deficits in autism spectrum disorder. Trends Neurosci. (2021) 44:793–807. doi: 10.1016/j.tins.2021.08.005, PMID: 34521563 PMC8484056

[43] van der Heijden ME Gill JS Sillitoe RV. Abnormal cerebellar development in autism Spectrum disorders. Dev Neurosci. (2021) 43:181–90. doi: 10.1159/000515189, PMID: 33823515 PMC8440334

[44] Ecker C Schmeisser MJ Loth E Murphy DG. Neuroanatomy and neuropathology of autism Spectrum disorder in humans. Adv Anat Embryol Cell Biol. (2017) 224:27–48. doi: 10.1007/978-3-319-52498-6_2, PMID: 28551749

[45] Lee E Lee J Kim E. Excitation/inhibition imbalance in animal models of autism Spectrum disorders. Biol Psychiatry. (2017) 81:838–47. doi: 10.1016/j.biopsych.2016.05.011, PMID: 27450033

[46] Lee EJ Choi SY Kim E. NMDA receptor dysfunction in autism spectrum disorders. Curr Opin Pharmacol. (2015) 20:8–13. doi: 10.1016/j.coph.2014.10.007 25636159

[47] Yizhar O Fenno LE Prigge M Schneider F Davidson TJ O’Shea DJ. Neocortical excitation/inhibition balance in information processing and social dysfunction. Nature. (2011) 477:171–8. doi: 10.1038/nature10360, PMID: 21796121 PMC4155501

[48] Goncalves J Violante IR Sereno J Leitao RA Cai Y Abrunhosa A. Testing the excitation/inhibition imbalance hypothesis in a mouse model of the autism spectrum disorder: in vivo neurospectroscopy and molecular evidence for regional phenotypes. Mol Autism. (2017) 8:47. doi: 10.1186/s13229-017-0166-4, PMID: 28932379 PMC5605987

[49] Karayannis T Au E Patel JC Kruglikov I Markx S Delorme R. Cntnap4 differentially contributes to GABAergic and dopaminergic synaptic transmission. Nature. (2014) 511:236–40. doi: 10.1038/nature13248, PMID: 24870235 PMC4281262

[50] Bateup HS Johnson CA Denefrio CL Saulnier JL Kornacker K Sabatini BL. Excitatory/inhibitory synaptic imbalance leads to hippocampal hyperexcitability in mouse models of tuberous sclerosis. Neuron. (2013) 78:510–22. doi: 10.1016/j.neuron.2013.03.017, PMID: 23664616 PMC3690324

[51] Wei H Ding C Jin G Yin H Liu J Hu F. Abnormal glutamate release in aged BTBR mouse model of autism. Int J Clin Exp Pathol. (2015) 8:10689–97. PMID: 26617779 PMC4637594

[52] Silverman JL Oliver CF Karras MN Gastrell PT Crawley JN. AMPAKINE enhancement of social interaction in the BTBR mouse model of autism. Neuropharmacology. (2013) 64:268–82. doi: 10.1016/j.neuropharm.2012.07.013, PMID: 22801296 PMC3445667

[53] Nuzzo T Sekine M Punzo D Miroballo M Katane M Saitoh Y. Dysfunctional d-aspartate metabolism in BTBR mouse model of idiopathic autism. Biochim Biophys Acta Proteins Proteom. (2020) 1868:140531. doi: 10.1016/j.bbapap.2020.140531, PMID: 32853769

[54] Burket JA Benson AD Tang AH Deutsch SI. D-Cycloserine improves sociability in the BTBR T+ Itpr3tf/J mouse model of autism spectrum disorders with altered Ras/Raf/ERK1/2 signaling. Brain Res Bull. (2013) 96:62–70. doi: 10.1016/j.brainresbull.2013.05.003, PMID: 23685206 PMC5581963

[55] Eissa N Venkatachalam K Jayaprakash P Falkenstein M Dubiel M Frank A. The multi-targeting ligand ST-2223 with histamine H3 receptor and dopamine D2/D3 receptor antagonist properties mitigates autism-like repetitive behaviors and brain oxidative stress in mice. Int J Mol Sci. (2021) 22:1947. doi: 10.3390/ijms22041947, PMID: 33669336 PMC7920280

[56] Han S Tai C Jones CJ Scheuer T Catterall WA. Enhancement of inhibitory neurotransmission by GABAA receptors having alpha2,3-subunits ameliorates behavioral deficits in a mouse model of autism. Neuron. (2014) 81:1282–9. doi: 10.1016/j.neuron.2014.01.016, PMID: 24656250 PMC4079471

[57] Kneussel M Brandstatter JH Laube B Stahl S Muller U Betz H. Loss of postsynaptic GABA(a) receptor clustering in gephyrin-deficient mice. J Neurosci. (1999) 19:9289–97. doi: 10.1523/JNEUROSCI.19-21-09289.1999, PMID: 10531433 PMC6782938

[58] Nelson SB Valakh V. Excitatory/inhibitory balance and circuit homeostasis in autism Spectrum disorders. Neuron. (2015) 87:684–98. doi: 10.1016/j.neuron.2015.07.033, PMID: 26291155 PMC4567857

[59] Gogolla N Takesian AE Feng G Fagiolini M Hensch TK. Sensory integration in mouse insular cortex reflects GABA circuit maturation. Neuron. (2014) 83:894–905. doi: 10.1016/j.neuron.2014.06.033, PMID: 25088363 PMC4177076

[60] Defensor EB Pearson BL Pobbe RL Bolivar VJ Blanchard DC Blanchard RJ. A novel social proximity test suggests patterns of social avoidance and gaze aversion-like behavior in BTBR T+ tf/J mice. Behav Brain Res. (2011) 217:302–8. doi: 10.1016/j.bbr.2010.10.033, PMID: 21055421 PMC3124342

[61] Rhine MA Parrott JM Schultz MN Kazdoba TM Crawley JN. Hypothesis-driven investigations of diverse pharmacological targets in two mouse models of autism. Autism Res. (2019) 12:401–21. doi: 10.1002/aur.2066, PMID: 30653853 PMC6402976

[62] Kazdoba TM Hagerman RJ Zolkowska D Rogawski MA Crawley JN. Evaluation of the neuroactive steroid ganaxolone on social and repetitive behaviors in the BTBR mouse model of autism. Psychopharmacology. (2016) 233:309–23. doi: 10.1007/s00213-015-4115-7, PMID: 26525567 PMC4703522

[63] Yoshimura RF Tran MB Hogenkamp DJ Ayala NL Johnstone T Dunnigan AJ. Allosteric modulation of nicotinic and GABAA receptor subtypes differentially modify autism-like behaviors in the BTBR mouse model. Neuropharmacology. (2017) 126:38–47. doi: 10.1016/j.neuropharm.2017.08.029, PMID: 28842344

[64] Hagerman RJ Berry-Kravis E Hazlett HC Bailey DB Moine H Kooy RF. Fragile X syndrome. Nat Rev Dis Primers. (2017) 3:17065. doi: 10.1038/nrdp.2017.65 28960184

[65] Li J Pelletier MR Perez Velazquez JL Carlen PL. Reduced cortical synaptic plasticity and GluR1 expression associated with fragile X mental retardation protein deficiency. Mol Cell Neurosci. (2002) 19:138–51. doi: 10.1006/mcne.2001.1085, PMID: 11860268

[66] Bostrom CA Majaess NM Morch K White E Eadie BD Christie BR. Rescue of NMDAR-dependent synaptic plasticity in Fmr1 knock-out mice. Cereb Cortex. (2015) 25:271–9. doi: 10.1093/cercor/bht237, PMID: 23968838

[67] Hu H Qin Y Bochorishvili G Zhu Y van Aelst L Zhu JJ. Ras signaling mechanisms underlying impaired GluR1-dependent plasticity associated with fragile X syndrome. J Neurosci. (2008) 28:7847–62. doi: 10.1523/JNEUROSCI.1496-08.2008, PMID: 18667617 PMC2553221

[68] Lee HK Takamiya K He K Song L Huganir RL. Specific roles of AMPA receptor subunit GluR1 (GluA1) phosphorylation sites in regulating synaptic plasticity in the CA1 region of hippocampus. J Neurophysiol. (2010) 103:479–89. doi: 10.1152/jn.00835.2009, PMID: 19906877 PMC2807233

[69] Krueger DD Osterweil EK Chen SP Tye LD Bear MF. Cognitive dysfunction and prefrontal synaptic abnormalities in a mouse model of fragile X syndrome. Proc Natl Acad Sci USA. (2011) 108:2587–92. doi: 10.1073/pnas.1013855108, PMID: 21262808 PMC3038768

[70] Eadie BD Cushman J Kannangara TS Fanselow MS Christie BR. NMDA receptor hypofunction in the dentate gyrus and impaired context discrimination in adult Fmr1 knockout mice. Hippocampus. (2012) 22:241–54. doi: 10.1002/hipo.20890, PMID: 21049485

[71] Yau SY Bettio L Chiu J Chiu C Christie BR. Fragile-X syndrome is associated with NMDA receptor hypofunction and reduced dendritic complexity in mature dentate granule cells. Front Mol Neurosci. (2018) 11:495. doi: 10.3389/fnmol.2018.00495, PMID: 30705620 PMC6344420

[72] Yun SH Trommer BL. Fragile X mice: reduced long-term potentiation and N-methyl-D-aspartate receptor-mediated neurotransmission in dentate gyrus. J Neurosci Res. (2011) 89:176–82. doi: 10.1002/jnr.22546, PMID: 21162125

[73] Di J Li J O’Hara B Alberts I Xiong L Li J. The role of GABAergic neural circuits in the pathogenesis of autism spectrum disorder. Int J Dev Neurosci. (2020) 80:73–85. doi: 10.1002/jdn.10005, PMID: 31910289

[74] Paluszkiewicz SM Martin BS Huntsman MM. Fragile X syndrome: the GABAergic system and circuit dysfunction. Dev Neurosci. (2011) 33:349–64. doi: 10.1159/000329420, PMID: 21934270 PMC3254035

[75] D'Hulst C De Geest N Reeve SP Van Dam D De Deyn PP Hassan BA. Decreased expression of the GABAA receptor in fragile X syndrome. Brain Res. (2006) 1121:238–45. doi: 10.1016/j.brainres.2006.08.115, PMID: 17046729

[76] Braat S D’Hulst C Heulens I De Rubeis S Mientjes E Nelson DL. The GABAA receptor is an FMRP target with therapeutic potential in fragile X syndrome. Cell Cycle. (2015) 14:2985–95. doi: 10.4161/15384101.2014.989114, PMID: 25790165 PMC4827888

[77] Adusei DC Pacey LK Chen D Hampson DR. Early developmental alterations in GABAergic protein expression in fragile X knockout mice. Neuropharmacology. (2010) 59:167–71. doi: 10.1016/j.neuropharm.2010.05.002, PMID: 20470805

[78] El Idrissi A Ding XH Scalia J Trenkner E Brown WT Dobkin C. Decreased GABA(a) receptor expression in the seizure-prone fragile X mouse. Neurosci Lett. (2005) 377:141–6. doi: 10.1016/j.neulet.2004.11.087, PMID: 15755515

[79] Sabanov V Braat S D’Andrea L Willemsen R Zeidler S Rooms L. Impaired GABAergic inhibition in the hippocampus of Fmr1 knockout mice. Neuropharmacology. (2017) 116:71–81. doi: 10.1016/j.neuropharm.2016.12.010, PMID: 28012946

[80] Gantois I Vandesompele J Speleman F Reyniers E D’Hooge R Severijnen LA. Expression profiling suggests underexpression of the GABA(a) receptor subunit delta in the fragile X knockout mouse model. Neurobiol Dis. (2006) 21:346–57. doi: 10.1016/j.nbd.2005.07.017, PMID: 16199166

[81] Zheleznova NN Sedelnikova A Weiss DS. Function and modulation of delta-containing GABA(a) receptors. Psychoneuroendocrinology. (2009) 34:S67–73. doi: 10.1016/j.psyneuen.2009.08.010, PMID: 19766404 PMC2794972

[82] Cogram P Deacon RMJ Warner-Schmidt JL von Schimmelmann MJ Abrahams BS During MJ. Gaboxadol normalizes behavioral abnormalities in a mouse model of fragile X syndrome. Front Behav Neurosci. (2019) 13:141. doi: 10.3389/fnbeh.2019.00141, PMID: 31293404 PMC6603241

[83] Heulens I D’Hulst C Van Dam D De Deyn PP Kooy RF. Pharmacological treatment of fragile X syndrome with GABAergic drugs in a knockout mouse model. Behav Brain Res. (2012) 229:244–9. doi: 10.1016/j.bbr.2012.01.031, PMID: 22285772

[84] Leblond CS Nava C Polge A Gauthier J Huguet G Lumbroso S. Meta-analysis of SHANK mutations in autism Spectrum disorders: a gradient of severity in cognitive impairments. PLoS Genet. (2014) 10:e1004580. doi: 10.1371/journal.pgen.1004580, PMID: 25188300 PMC4154644

[85] SFARI (2022). SHANK3 [Online]. Available at: https://gene.sfari.org/database/animal-models/genetic-animal-models/SHANK3/Mus%20musculus#genetic-models-tab (Accessed October 20, 2022).

[86] Heise C Preuss JM Schroeder JC Battaglia CR Kolibius J Schmid R. Heterogeneity of cell surface glutamate and GABA receptor expression in shank and CNTN4 autism mouse models. Front Mol Neurosci. (2018) 11:212. doi: 10.3389/fnmol.2018.00212, PMID: 29970989 PMC6018460

[87] Wang X McCoy PA Rodriguiz RM Pan Y Je HS Roberts AC. Synaptic dysfunction and abnormal behaviors in mice lacking major isoforms of Shank3. Hum Mol Genet. (2011) 20:3093–108. doi: 10.1093/hmg/ddr212, PMID: 21558424 PMC3131048

[88] Bukatova S Renczes E Reichova A Filo J Sadlonova A Mravec B. Shank3 deficiency is associated with altered profile of neurotransmission markers in pups and adult mice. Neurochem Res. (2021) 46:3342–55. doi: 10.1007/s11064-021-03435-6, PMID: 34453663

[89] Horder J Andersson M Mendez MA Singh N Tangen A Lundberg J. GABAA receptor availability is not altered in adults with autism spectrum disorder or in mouse models. Sci Transl Med. (2018a) 10:eaam8434. doi: 10.1126/scitranslmed.aam8434 30282698

[90] Blatt GJ Fatemi SH. Alterations in GABAergic biomarkers in the autism brain: research findings and clinical implications. Anat Rec. (2011) 294:1646–52. doi: 10.1002/ar.21252, PMID: 21901839 PMC3190183

[91] Fatemi SH Reutiman TJ Folsom TD Rooney RJ Patel DH Thuras PD. mRNA and protein levels for GABAAalpha4, alpha5, beta1 and GABABR1 receptors are altered in brains from subjects with autism. J Autism Dev Disord. (2010) 40:743–50. doi: 10.1007/s10803-009-0924-z, PMID: 20066485 PMC2865581

[92] Fatemi SH Reutiman TJ Folsom TD Thuras PD. GABA(a) receptor downregulation in brains of subjects with autism. J Autism Dev Disord. (2009) 39:223–30. doi: 10.1007/s10803-008-0646-7, PMID: 18821008 PMC2697059

[93] Oblak A Gibbs TT Blatt GJ. Decreased GABAA receptors and benzodiazepine binding sites in the anterior cingulate cortex in autism. Autism Res. (2009) 2:205–19. doi: 10.1002/aur.88, PMID: 19650112 PMC2762426

[94] Oblak AL Gibbs TT Blatt GJ. Reduced GABAA receptors and benzodiazepine binding sites in the posterior cingulate cortex and fusiform gyrus in autism. Brain Res. (2011) 1380:218–28. doi: 10.1016/j.brainres.2010.09.021, PMID: 20858465 PMC3020259

[95] Guptill JT Booker AB Gibbs TT Kemper TL Bauman ML Blatt GJ. [^3^H]-flunitrazepam-labeled benzodiazepine binding sites in the hippocampal formation in autism: a multiple concentration autoradiographic study. J Autism Dev Disord. (2007) 37:911–20. doi: 10.1007/s10803-006-0226-7, PMID: 17019626

[96] Blatt GJ Fitzgerald CM Guptill JT Booker AB Kemper TL Bauman ML. Density and distribution of hippocampal neurotransmitter receptors in autism: an autoradiographic study. J Autism Dev Disord. (2001) 31:537–43. doi: 10.1023/a:1013238809666, PMID: 11814263

[97] Behuet S Cremer JN Cremer M Palomero-Gallagher N Zilles K Amunts K. Developmental changes of glutamate and GABA receptor densities in Wistar rats. Front Neuroanat. (2019) 13:100. doi: 10.3389/fnana.2019.00100, PMID: 31920569 PMC6933313

[98] D'Hulst C Heulens I Van der Aa N Goffin K Koole M Porke K. Positron emission tomography (PET) quantification of GABAA receptors in the brain of fragile X patients. PLoS One. (2015) 10:e0131486. doi: 10.1371/journal.pone.0131486, PMID: 26222316 PMC4519313

[99] Mendez MA Horder J Myers J Coghlan S Stokes P Erritzoe D. The brain GABA-benzodiazepine receptor alpha-5 subtype in autism spectrum disorder: a pilot [(11)C]Ro15-4513 positron emission tomography study. Neuropharmacology. (2013) 68:195–201. doi: 10.1016/j.neuropharm.2012.04.008, PMID: 22546616 PMC4489617

[100] Mori T Mori K Fujii E Toda Y Miyazaki M Harada M. Evaluation of the GABAergic nervous system in autistic brain: (123)I-iomazenil SPECT study. Brain Dev. (2012) 34:648–54. doi: 10.1016/j.braindev.2011.10.007, PMID: 22099869

[101] Brondino N Fusar-Poli L Panisi C Damiani S Barale F Politi P. Pharmacological modulation of GABA function in autism spectrum disorders: a systematic review of human studies. J Autism Dev Disord. (2016) 46:825–39. doi: 10.1007/s10803-015-2619-y, PMID: 26443675

[102] Filice F Janickova L Henzi T Bilella A Schwaller B. The Parvalbumin hypothesis of autism spectrum disorder. Front Cell Neurosci. (2020a) 14:577525. doi: 10.3389/fncel.2020.577525, PMID: 33390904 PMC7775315

[103] Lawrence YA Kemper TL Bauman ML Blatt GJ. Parvalbumin-, calbindin-, and calretinin-immunoreactive hippocampal interneuron density in autism. Acta Neurol Scand. (2010) 121:99–108. doi: 10.1111/j.1600-0404.2009.01234.x, PMID: 19719810

[104] Hashemi E Ariza J Rogers H Noctor SC Martinez-Cerdeno V. The number of Parvalbumin-expressing interneurons is decreased in the prefrontal cortex in autism. Cereb Cortex. (2017) 27:1931–43. doi: 10.1093/cercor/bhw021, PMID: 26922658 PMC6074948

[105] Wohr M Orduz D Gregory P Moreno H Khan U Vorckel KJ. Lack of parvalbumin in mice leads to behavioral deficits relevant to all human autism core symptoms and related neural morphofunctional abnormalities. Transl Psychiatry. (2015) 5:e525. doi: 10.1038/tp.2015.19, PMID: 25756808 PMC4354349

[106] Briones BA Pisano TJ Pitcher MN Haye AE Diethorn EJ Engel EA. Adult-born granule cell mossy fibers preferentially target parvalbumin-positive interneurons surrounded by perineuronal nets. Hippocampus. (2021) 31:375–88. doi: 10.1002/hipo.23296, PMID: 33432721 PMC8020456

[107] Filice F Vorckel KJ Sungur AO Wohr M Schwaller B. Reduction in parvalbumin expression not loss of the parvalbumin-expressing GABA interneuron subpopulation in genetic parvalbumin and shank mouse models of autism. Mol Brain. (2016) 9:10. doi: 10.1186/s13041-016-0192-8, PMID: 26819149 PMC4729132

[108] Selby L Zhang C Sun QQ. Major defects in neocortical GABAergic inhibitory circuits in mice lacking the fragile X mental retardation protein. Neurosci Lett. (2007) 412:227–32. doi: 10.1016/j.neulet.2006.11.062, PMID: 17197085 PMC1839948

[109] Culotta L Penzes P. Exploring the mechanisms underlying excitation/inhibition imbalance in human iPSC-derived models of ASD. Mol Autism. (2020) 11:32. doi: 10.1186/s13229-020-00339-0, PMID: 32393347 PMC7216514

[110] Filice F Schwaller B Michel TM Grunblatt E. Profiling parvalbumin interneurons using iPSC: challenges and perspectives for autism spectrum disorder (ASD). Mol Autism. (2020b) 11:10. doi: 10.1186/s13229-020-0314-0, PMID: 32000856 PMC6990584

